# Exploring Blue Light Inactivation of Foodborne Viruses on Strawberries During Washing

**DOI:** 10.3390/foods15203728

**Published:** 2026-10-20

**Authors:** Macray Metz, Ilana Torres-Shannon, Issmat I. Kassem, Francisco Diez-Gonzalez, Malak A. Esseili

**Affiliations:** Center for Food Safety, Department of Food Science and Technology, University of Georgia, 1109 Experiment Street, Griffin, GA 30223, USA

**Keywords:** human norovirus, hepatitis A virus, blue light, strawberries, washing

## Abstract

Human norovirus (HuNoV) and hepatitis A virus (HAV) are the main enteric viruses causing foodborne viral outbreaks. Frozen berries have been implicated in multi-state and international outbreaks attributed to these viruses. Because strawberries undergo washing immediately prior to freezing, supplementary non-thermal interventions are needed during this step to enhance viral inactivation. The objective of this study was to explore the use of blue light (405 nm) inactivation of foodborne viruses on strawberries during washing. Because infectious HuNoV is difficult to generate in cell culture, Tulane virus (TV) was used as a surrogate. Strawberries were contaminated with TV or HAV then submerged in sterile water under gentle shaking, with or without BL treatment (~1000 J/cm^2^) at room temperature. A modified FDA/BAM/26 method was used to recover infectious viruses, and the infectivity titers were quantified using the 50% tissue culture infectious dose (TCID_50_) assay. Indigenous bacteria on strawberries and in the wash water and the water physiochemical parameters were monitored. The combined effect of washing strawberries under blue light (~1000 J/cm^2^) resulted in ~1.7 and 1.5 log reduction for TV and HAV on strawberries and 1 log reduction in wash water, respectively. Bacteria count on washed strawberries or in wash water were not impacted by BL. Blue light significantly increased the oxidizing potential of the wash water while not impacting the pH, turbidity, or temperature of the water. In conclusion, this study demonstrates that BL treatment during strawberry washing can reduce the viral load on strawberries and in wash water. Further optimizations will be critical to adapt this technology to enhance strawberry safety, quality and prevent cross-contamination during the washing process.

## 1. Introduction

Human norovirus (HuNoV) is the leading cause of acute gastroenteritis worldwide [1]. The virus is estimated to cause 5.5 million cases of foodborne illnesses within the US alone [2] and 685 million cases of acute gastroenteritis worldwide [1]. Despite the public health impact, there are still no licensed vaccines, antivirals, or alternative therapies for HuNoV, but there are some vaccine candidates in clinical trials [3]. The 50% infectious dose (ID50) for HuNoV is estimated as low as ~18 viral particles [4] to 2800 genomic equivalent [5]. Hepatitis A virus is the leading cause of acute hepatitis, which can progress to liver failure in immunocompromised people [6]. Globally, there has been a 13.9% surge in HAV infection cases from 139 million in 1990 to 158 million in 2019 [7]. However, there are vaccines that can be used to prevent HAV infection [8]. HuNoV and HAV are fecal-orally transmitted pathogens which can contaminate a wide variety of matrices including fomites, water, and food [9]. Annually, HuNoV causes an estimated $4.2 billion in direct healthcare costs, and $56.1 billion in lost productivity, resulting in a total global societal economic burden of $60.3 billion [10]. In the case of HAV infections within the US, the average cost per hospitalization was estimated at approximately $16,000 and the cost of out-patient was approximately $2520 dollars [11]. Between 2016 and 2020, the total estimated cost of HAV-associated hospitalization in the US was $306.8 million [12].

Because it is difficult to culture HuNoV, surrogate viruses are commonly used. Several HuNoV surrogates such as murine norovirus (MNV), feline calicivirus (FCV), porcine sapovirus, Tulane virus (TV) and bacteriophages such as MS2 have been used. Tulane virus mimics HuNoV attachment to cells via histo-blood group antigens, and while classified in a separate genus, it is closely related to HuNoV within the same family [13]. Tulane virus was originally isolated from the stools of rhesus macaques [14] and can replicate in rhesus monkey kidney cells, LLC-MK2 [15], which are used to quantify the virus infectious titer.

Berries are widely consumed for their health-promoting properties [16]. However, because berries undergo minimal processing from farm-to-fork, contamination with fecal pathogens is of high concern [17]. In terms of fresh produce, fresh berries were implicated in 2% of outbreaks within the US and 51% of outbreaks within the EU [18]. Globally, 68 total virus outbreaks associated with berries occurred between 1983 and 2018, in which HuNoV caused 46 outbreaks. Of the 68 outbreaks associated with berries, frozen berries were implicated in 50 outbreaks, of which 72% were associated with HuNoV and 28% were associated with HAV [19]. The largest HuNoV outbreak associated with berries occurred in Germany in 2012. The outbreak was traced to frozen strawberries imported from China, and it impacted 390 facilities, with ~11,000 cases of gastroenteritis and 38 hospitalizations [20]. In the US, a recent outbreak of HAV linked to berries was traced to imported frozen strawberries from Mexico in which 10 people got sick and 4 people were hospitalized [21]. Freezing strawberries has minimal impact on the infectivity of these viruses [22]. Thus, there is an urgent need for better control measures on the farm and during minimal processing to prevent and reduce virus contamination of berries.

Strawberries are subjected to minimal processing before being frozen. One such processing method is washing to remove debris, but it can also act as a means of reducing pathogenic or spoilage microbes when a sanitizer is included [23]. On the industrial level, strawberries intended for freezing are typically washed using gentle agitation, either with or without an added sanitizer [23]. Several studies investigated the effect of washing to remove viruses from berries. A previous study found that coliphage MS2 spiked on strawberries was reduced by 68% (0.5 log) when subjected to a water wash treatment for 12 s; however, when 200 ppm of free chlorine was added, there was 96% (1.5 log) virus reduction after 12 s [23]. One study showed that mimicking consumers’ washing of strawberries in potable water for 2 min resulted in a 1 log reduction for infectious HAV [24]. Similarly, a study found that washing strawberries with 50 ppm of chlorine in potable water for 2 min resulted in infectious viral reduction of 2.1, 1.5, and 3.4 for MS2, MNV, and HAV, respectively [24]. Lastly, a previous study reported that washing MNV-contaminated strawberries with 200 ppm of chlorine for 2 min resulted in a 1 log reduction of infectious virus. However, under the same conditions washing the contaminated strawberries in just regular tap water caused a 0.8 log reduction of infectious MNV [25]. Taken together, washing berries with just water can remove ~0.5–1 log of infectious viruses, while addition of chlorine (50–200 ppm) increased viral reduction to ~1.5–2.1 log for MS2, 1–1.5 log for MNV and 3.4 log for HAV. A drawback to using chemical sanitizer such as chlorine to inactivate viruses on strawberries is the generation of carcinogenic compounds such as trihalomethanes (THMs) and haloacetonitriles (HANs) [26]. These compounds are known to have toxic effects on the kidneys and the liver [26]. For this reason, several European countries have banned the use of chlorine in produce washing [27]. Coupled with the toxicity issues, the presence of organic matter in the wash water impedes the effectiveness of chlorine [28]. Thus, there is a need for alternative treatments of contaminated berries during washing. 

Light-based technologies for the inactivation of microorganisms have been studied across both the visible and the ultraviolet (UV) spectrums [29,30]. In spite of its known antimicrobial action, UV light has notable limitations, including low penetration depth, the risk of skin cancer (melanomas), and ocular damage [31]. An alternative light-based technology, blue light (BL)—a visible light with a peak wavelength between 380–500 nm—has recently gained attention for its antimicrobial effect. In natural freshwater, BL has the greatest penetration depth compared to any other visible wavelength, reaching depths of up to 1000 m in clear water [32]. One of the biggest hurdles for the use of light-based technology is achieving uniform light distribution while in suspension. The samples have to undergo continuous agitation to ensure proper uniform light distribution across the samples [33]. The irradiance of BL is a function of both the wavelength and distance to the sample [34]. By measuring the irradiance with a spectroradiometer, the resultant dose (J/cm^2^) is calculated by multiplying the irradiance of BL by the time of exposure (in seconds) of the sample. One of the main reasons for the use of BL is that it is much less detrimental to mammalian cells than UV light [35]. However, prolonged BL exposure can induce photooxidative damage [36].

The antimicrobial properties of BL stem from the absorption of these wavelengths by porphyrins within bacteria, leading in the presence of dissolved oxygen to the photochemical production of reactive oxygen species (ROS) [37]. These endogenous intracellular porphyrins feature conjugated ring structures that readily absorb photons [38]. Reactive oxygen species are typically short lived and do not strictly need water around, but water helps facilitate the resultant mechanism of action. The ROS results in non-specific oxidative damage to bacterial structures, thus causing inactivation. The bactericidal properties of BL have been investigated for a plethora of microorganisms and with various matrices including liquids and food [30]. However, there is a limited number of studies on the effect of BL on viruses, let alone on fruits within water systems. Because viruses lack endogenous porphyrins, exogenous chromophores can be introduced as photosensitizers that absorb light to generate ROS [39]. Photosensitizers are chemicals that have a multi-ring structure similar to that of porphyrin rings that could be used to generate ROS [40]. Additionally, photosensitizers naturally present in surrounding organic matter can be exploited to inactivate viruses upon blue light exposure [41].

There are few studies investigating the effect of BL on viruses in food or environmental matrices. For enveloped viruses, BL treatment seems to produce high inactivation at a relatively lower dose. For example, BL (405 nm) with doses of ~57.6 and 86.4 J/cm^2^ inactivated feline infectious peritonitis virus (an enveloped coronavirus virus) on inanimate surfaces (metal, paper, and plastic) by 4.2 and 4.5 log reduction of TCID_50_/mL, respectively [42]. In another study, BL (420 nm,108 J/cm^2^) caused a greater than 4 log reduction of infectious SARS-CoV-2 (an enveloped virus) suspended in cell culture media (DMEM) [37]. For non-enveloped viruses, the effect of BL seemed variable and required a higher dose. For example, it was reported that treatment with BL (405 nm, 2800 J/cm^2^) resulted in a 4 log reduction for FCV suspended in DMEM [41]. Another study examined the effects of BL 405 nm at 4.2 mWcm^−2^ at a distance of ~7.5 cm (7.56 J/cm^2^) against TV spiked on blueberries under cold conditions with no washing. The authors found that BL alone caused a 0.1 log reduction and that the addition of either riboflavin or rose bengal at 0.1% caused a 0.5 or 1 log reduction, respectively [39]. 

There are a couple studies on bacteriophages which are sometimes used as surrogates for human pathogenic viruses. For example, BL exposure (455 nm, 7200 J/cm^2^) resulted in more than 3 log reduction in infectivity titer for an enveloped bacteriophage Phi6 suspended in PBS solution [43]. In another study, BL treatment (405 nm, ~1400 J/cm^2^) resulted in 7 log reduction for bacteriophage ΦC31, a surrogate for double stranded non-enveloped DNA viruses, when suspended in nutrient broth [44]. Another study used bacteriophage MS2 on surfaces and found that the surface type influenced log reduction. Specifically, a 3.9 log reduction was reported for MS2 on polytetrafluorethylene, but only a 0.3 log reduction was found on ceramic surfaces, when BL (405 nm) was used at a dose of 2804 J/cm^2^ [34]. Therefore, BL appears to be an emerging antiviral treatment that could potentially be used to inactivate a wide range of viruses. However, majority of previous studies used high-intensity BL lamps that generate undesirable heat on solid surfaces, such as ceramic, polytetrafluoroethylene, or fresh blueberries, thereby requiring a cooling system [34,39,42]. Even though liquid suspension tolerates high intensities much better [37,44], this capacity is finite; exceeding a certain intensity threshold still necessitates active cooling to prevent excessive temperature increases [41,43]. 

To date, very few studies have investigated the use of BL in food industry-relevant scenarios, highlighting a critical need for research on its antiviral effects in water and food matrices. Therefore, we explored the use of BL (405 nm at approx. 1000 J/cm^2^) to inactivate foodborne viruses on strawberries during washing at room temperature. The low intensity BL lamps tested in this study are inexpensive, energy-efficient, do not require thermal management and can be applied on a continuous basis in resource-limited settings. While this exploratory study evaluated only a single BL dose, the results demonstrate the baseline feasibility of the system.

## 2. Materials and Methods

### 2.1. Cell Culture Passage

For FRhK-4 (CRL-1688 ^TM^, ATCC, Manassas, VA, USA) and LLC-MK2 (CCL-7 ^TM^, ATCC) cell lines, growth media consisted of Dulbecco’s Modified Eagle medium (DMEM) (VWR, Radnor, PA, USA) or medium 199 (M199) (Fisher Scientific, Waltham, MA, USA), supplemented with either 10% or 5% fetal bovine serum (FBS) (Fisher Scientific), respectively along with 1% antibiotic-antimycotic (anti-anti) (Fisher Scientific). These cells were routinely passaged in T175 cell culture flasks (Fisher Scientific). Briefly, cells were washed using Dulbecco’s phosphate buffered saline (DPBS) (Fisher Scientific), trypsinized to passage them into new flasks and incubated inside a 5% CO_2_ incubator at 37 °C until cells reached confluency (within 3–5 days) [45]. 

### 2.2. Virus Stocks

Tulane virus and HAV (HM175/18f) (ATCC, Gaithersburg, MD, USA) stocks were prepared by infecting LLC-MK2 and FRhK-4 cells, respectively as described previously [45,46]. Specifically, the cells were cultured until they reached ~90% confluency in the T175 flasks. The virus seed stock was added to the infection media which is prepared with respective cell culture media supplemented with 2% FBS, and 1% anti-anti. The old media was removed from the T175 flasks, and the cells were washed once with DPBS. The infection media containing the viruses were added to the respective flasks. The flasks were incubated until ~80% CPE was observed, which usually occurs within 2–3 and 6–7 days for TV and HAV, respectively. The flasks were then frozen and subjected to three rounds of freeze/thawing. The cells were scrapped from the surface and centrifuged at 100× *g* for 10 min at 4 °C. The supernatants were ultra-filtered using Amicon 100 kDa Ultra-15 centrifugal filter tubes (Millipore, Burlington, MA, USA) at 100× *g* for 30 min to semi-purify the viruses and concentrate them by a factor of 10×. 

### 2.3. Virus Inoculations on Strawberries

Fresh strawberries were purchased from local grocery stores. Firm undamaged strawberries were selected, and the calyx was removed from each strawberry before use. Frozen stocks of viruses (TV and HAV) were thawed on ice and diluted as needed. Tulane virus and HAV were each spot inoculated separately on different strawberry replicates (~10 g/replicate) as 10 μL droplets for a final volume of 100 μL. The viral spots were allowed to completely dry (usually within ~45 min) and then the strawberries to be washed were placed inside glass beakers.

### 2.4. The Effect of Blue Light on Foodborne Viruses on Strawberries During Washing

To explore the effect of BL on viruses while washing the virus-contaminated strawberries, low intensity 405 nm light-emitting diode (LED) array lamps (FASTTOBUY Resin Curing Light 405 nm with 20 W Power, FASTTOBUY, Shenzhen, China) were fixed at 4.5 inches above the glass beakers containing the virus-spiked strawberries, ensuring the light was hitting each sample. The glass beakers were placed on a rotational shaker set at 150 rpm to mimic gentle washing. Each strawberry replicate was completely submerged in 30 mL of sterile Milli-Q water inside a 50-mL glass beaker. Sterile water was used to reduce variation between independent experiments and to be able to attribute any observed effects in water to BL. One set of beakers containing strawberries (n = 3) was wrapped in aluminum foil to prevent BL from getting through while the other set was exposed to BL. Another set of beakers (n = 3) containing strawberry replicates was used as a control positive to determine virus recovery without any washing or BL exposure. Also, a water control positive was included, whereby 100 μL of viruses (TV or HAV) were spiked into 30 mL of sterile milli-q water to determine recovery of virus from water. The irradiance (~70 mW/cm^2^) was measured using HÖnle UV Meter (HÖnle UV Technology, Gilching, Germany) to calculate the BL dose in J/cm^2^ unit [47]. Then, the shaker was turned on and allowed to shake at 150 rpm for 4 h to generate a fixed BL exposure dose of ~1000 J/cm^2^.

Once the BL treatment was done, the strawberries were removed from the water. The strawberries displayed no signs of discoloration or damage. From the strawberry wash water samples, one mL was saved for quantification of bacteria in water. Also, the water physiochemical parameters were measured using a Hanna probe (HI9829, Hanna instruments, Woonsocket, RI, USA) to determine pH, oxidation reduction potential, dissolved oxygen, conductivity, resistivity, turbidity, and temperature of the samples. The water samples were subjected to ultracentrifugation using Optima XE-90 (Beckman Coulter, Brea, CA, USA) ultracentrifuge at 175,000× *g* for 45 min at 4 °C. The resulting pellets containing concentrated viruses were re-suspended in 1 mL of PBS.

Washed strawberries were processed through the FDA/BAM/26 method as described below. The wash water as well as the strawberries samples were subjected to infectivity assays for TV or HAV as described below.

### 2.5. Recovery of Infectious Viruses from Strawberries

Each strawberry replicate was processed using the FDA method (BAM chapter 26, section A2) [48] with slight modification to the elution buffer pH and percent beef extract to allow better recovery of infectious viruses (referred to as mBAM26), as shown in our previous research [49]. This mBAM26 had an infectivity LOD of 1.6 to 1.8 log TCID_50_/50 g and a recovery efficiency of 76.6 ± 26.1% [49]. In short, each strawberry replicate was added to a whirl-pack bag containing 30 mL of Tris-glycine beef extract, 1% TGBE, pH 8 instead of 6% TGBE, pH 9.5 supplemented with 50 μL of pectinase (2500 U/mL). A negative control bag containing non-inoculated strawberries received 100 μL of PBS. Each bag was inverted three times and allowed to shake at 150 rpm for 15 min at room temperature. The resulting eluants were transferred to 50-mL falcon tubes. One mL from the strawberry eluants was saved for ~1–2 h at 4 °C for quantification of indigenous bacteria as described below. Using Avanti J-15R and JA-10.100 Fixed Angle Rotor (Beckman Coulter, Brea, CA, USA), all falcon tubes underwent centrifugation at 10,000× *g* for 15 min at 4 °C. The pH of the control negative samples was monitored to make sure the pH is ~8. All supernatants were transferred to ultra-clear thin-walled ultracentrifugation tubes (Beckman Coulter, Brea, CA, USA). The ultracentrifuge tubes were balanced with one another within 0.01 g using PBS. Using Optima XE-90 ultracentrifuge (Beckman Coulter, Brea, CA, USA), the tubes were spun at 175,000× *g* for 45 min at 4 °C. Once ultracentrifugation is completed, the pellets were resuspended in 1 mL of PBS. From the recovered virus samples, 250 μL were saved for TCID_50_ testing as described below. All samples were saved at −80 °C for further testing. 

### 2.6. Infectivity Assays (TCID_50_)

For HAV and TV infectivity assays, cell lines were plated as 8 × 10^4^ and 2.6 × 10^5^ of LLC-MK2 and FRhK-4 cells, respectively into 96-well tissue culture treated, flat-bottom microplates (Thermo Fisher Scientific, Waltham, MA, USA). The cells were allowed to grow until they reached 95–100% confluency, typically within 5–7 days for LL-CMK2 and 7–10 days for FRhK-4 cells. On the day of testing the samples, the media was replaced with infectivity media, (same as growth media but with 2% FBS). A serial dilution of the sample containing viruses was done ranging from undiluted to 10^−5^. Then, 50 μL of each sample dilution was pipetted into four well containing the cells. The plates were incubated inside a 5% CO_2_ incubator at 37 °C for 1–2 h and then the first 2 rows were replaced with fresh infection media to reduce any cytotoxic effect from the strawberry sample itself. The plates were incubated for 7–10 days (HAV) or 5–7 days (TV) before observation of CPE. Viral titers were estimated following the Reed-Muench equation for the calculation of TCID_50_ [50]. Each infected set of plates had a control positive (virus with known titer) and control negative (cell culture media).

### 2.7. Statistical Analysis

Each BL-strawberry washing experiment was conducted independently three times for HAV and TV separately. Each virus experiment contained three biological replicates for each treatment. A biological replicate is one strawberry (~10 g) or one volume of water (30 mL). The entire virus and bacteria data sets were log_10_-transformed to calculate log reductions. Mean values and standard deviations were derived from all biological replicates (n = 9). GraphPad Prism 11.1.0 was used for all statistical analyses. *T*-tests were used for comparing two means and ANOVA followed by Tukey’s test were used for multiple comparisons of means. Correlation analyses and multiple linear regression analyses were used to determine relationships between water physiochemical parameters and the combined virus infectivity titers (n = 18), or bacteria counts with and without BL treatments. Statistical significance was defined as *p* < 0.05.

## 3. Results

### 3.1. Effect of Blue Light Treatment on Infectious Viruses on Strawberries During Washing

Fresh strawberries were contaminated with TV or HAV, allowed to dry, and then left unwashed (control positive) or washed in sterile water with or without BL (405 nm, 1000 J/cm^2^) treatment. For TV and HAV, an average of 4.1 ± 0.4 and 3.9 ± 0.4 log TCID_50_/10 g, respectively were recovered from control positive strawberry samples. From spiked positive water samples not subjected to any treatments, an average of 3.8 ± 0.4 and 3.5 ± 0.3 log TCID_50_/mL were recovered for TV and HAV, respectively. There were no significant differences between the two viruses’ infectivity titers on strawberries or in water.

**Figure 1:**
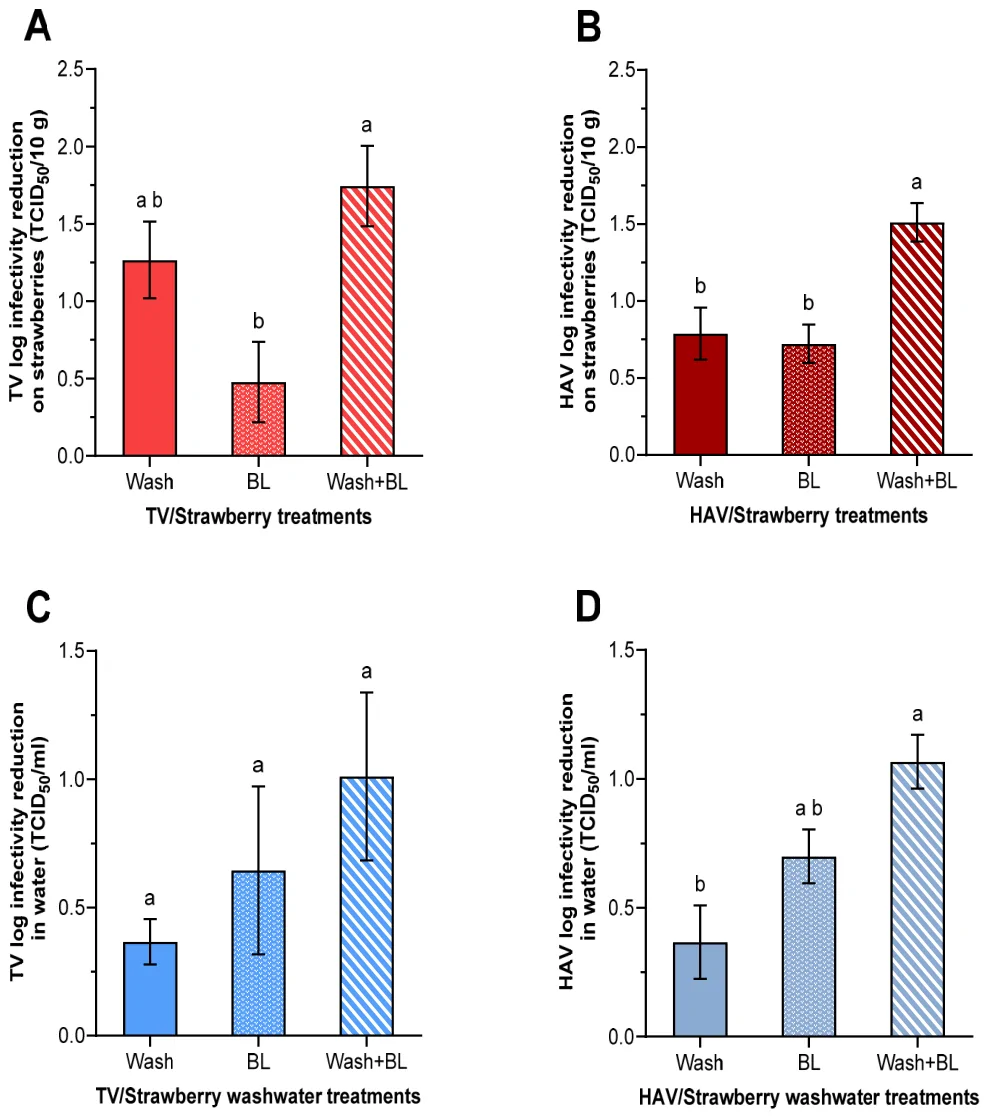
Infectious virus log reduction in response to blue light (405 nm, dose 1000 J/cm^2^) treatment of virus-contaminated strawberries. Strawberries underwent washing in sterile water while being exposed or unexposed to blue light. Viruses (**A**) Tulane virus and (**B**) hepatitis A virus on strawberries or in the corresponding wash water (**C**) Tulane virus and (**D**) hepatitis A virus that were either exposed to blue light or not. Means with different letters indicate significant differences (*p* < 0.05).

The effect of shaking strawberries in water (i.e., washing alone) resulted in ~1.2 log reduction in infectious TV on strawberries, while treatment with BL alone resulted in ~0.5 log reduction; however, the combined effect of washing and BL treatment caused the largest reduction of 1.7 log (Figure 1A) (*p* < 0.05). For HAV, washing or BL alone caused a reduction of infectious HAV on strawberries by 0.7 log, while the combined effect caused a significant reduction of 1.5 log (Figure 1B). For both viruses, the reduction caused by BL alone was not significantly different from the reduction caused by washing alone. Therefore, the combined effect of washing strawberries under BL (405 nm, 1000 J/cm^2^) treatment was most effective in inactivating infectious viruses, resulting in 96.9–98.2% reductions.

### 3.2. Effect of Blue Light Treatment on Infectious Viruses in Strawberry Wash Water

Following the end of strawberry washing period, infectious viruses were quantified in the recovered wash water samples. Under BL exposure, the combined effect of BL and washing (i.e., shaking) in water resulted in 1.01 log reduction in infectivity titer for TV in wash water (Figure 1A). Although this was higher than the inactivation of TV in wash water due to BL alone or wash alone, the difference was not statistically significant (Figure 1C). A similar effect was observed for HAV, whereby, the combined effect of washing and BL, caused a reduction in infectious HAV in wash water by 1.06 log (Figure 1D). However, for HAV, the combined effect of BL and washing was significantly different from the effect of washing alone (Figure 1D). Regardless of washing, BL alone resulted in 0.6 and 0.7 log reduction in wash water, for TV and HAV, respectively (Figure 1C,D).

### 3.3. Effect of Blue Light Treatment on Strawberries’ Indigenous Bacteria During Washing

**Figure 2:**
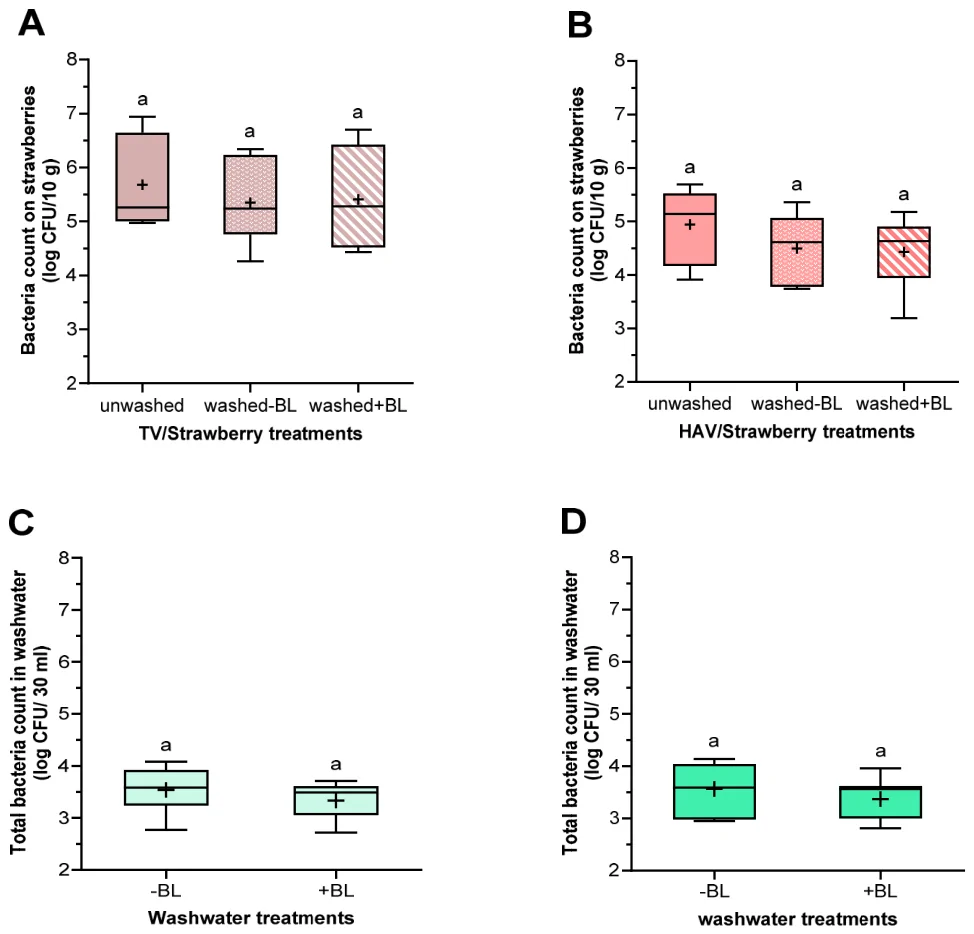
Effect of blue light (405 nm, dose 1000 J/cm^2^) on indigenous bacteria of strawberries that underwent washing in sterile water while being exposed to BL. Bacteria were quantified from both (**A**) Tulane virus and (**B**) hepatitis A virus -contaminated strawberries on strawberries and in wash water (**C**,**D**). Means indicated with (+) that have different letters indicate significant differences (*p* < 0.05).

Initial heterotrophic bacteria population count on fresh strawberries brought from local grocery stores and used for various experiments varied widely between trials. The strawberries contaminated with TV had an average bacteria count of 5.6 ± 0.8 log CFU/10 g (Figure 2A). However, washing with or without exposure to BL (5.3 and 5.4 log CFU/10 g, respectively) had no significant effects on indigenous bacteria counts on strawberries as compared to initial counts (Figure 2A). For HAV trials, the initial average bacteria count on strawberries was 4.9 ± 0.6 log CFU/10 g (Figure 2B). Although this count had a slight decrease upon treatment of strawberries with BL under washing (~4.4 log CFU/10 g), the difference was not statistically significant (Figure 2B). Therefore, BL had no significant effect on indigenous bacteria on strawberries during washing.

Sterile water was used to submerge the strawberries; however, by the end of the washing period, the bacterial population in wash water collected from the beakers was 3.3 and 3.5 log CFU/mL, with or without BL treatment, respectively (Figure 2A,B). Thus, BL had no significant effects on the bacteria washed off the strawberries into water.

### 3.4. Effect of Blue Light Treatment on the Physiochemical Parameters of the Strawberry Wash Water

**Figure 3:**
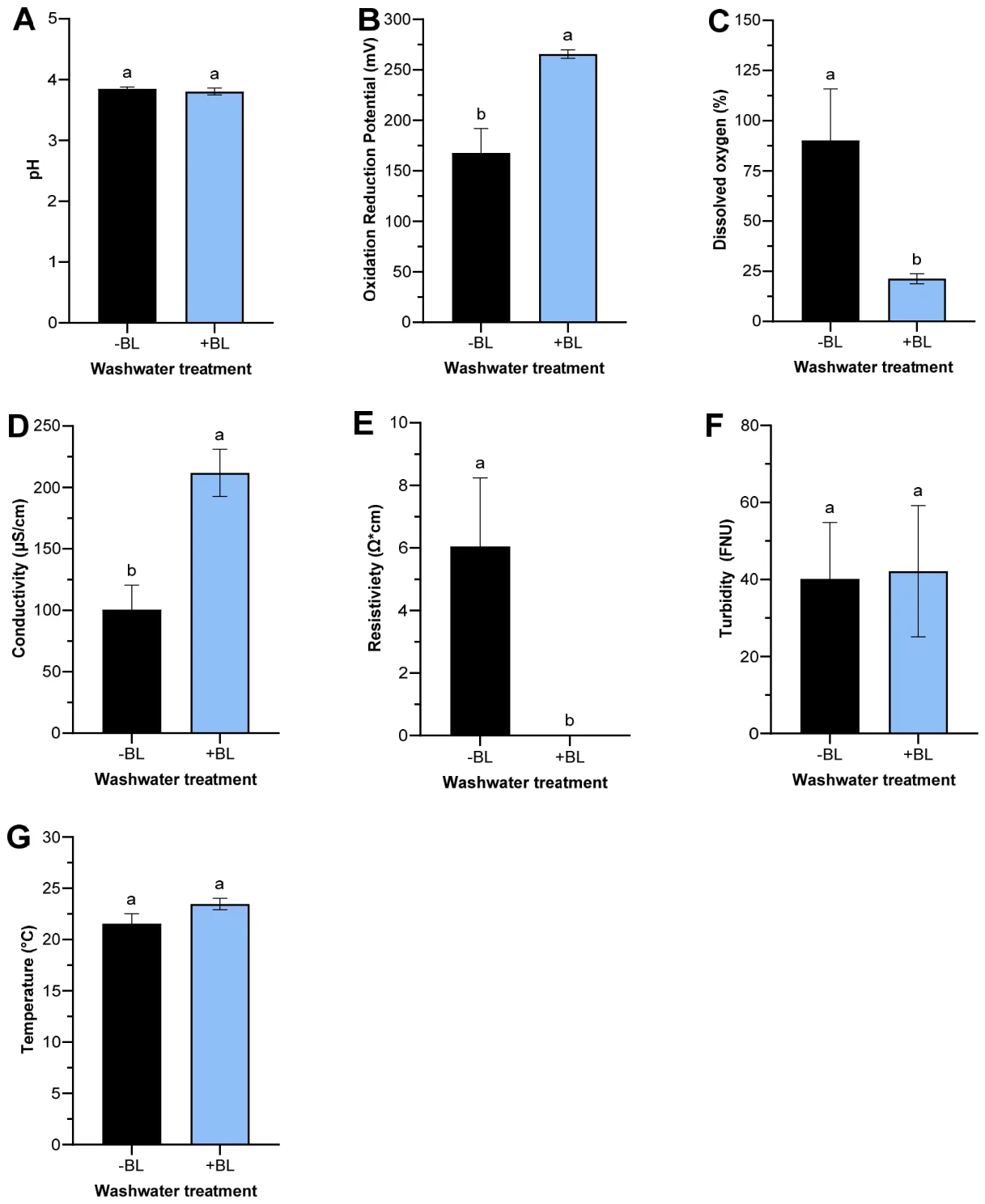
Effect of blue light (405 nm) on strawberry wash water physiochemical properties. (**A**–**G**) are the water physiochemical properties with (**A**) pH, (**B**) oxidation reduction potential, (**C**) dissolved oxygen, (**D**) conductivity, (**E**) Resistivity, (**F**) turbidity, and (**G**) temperature. Means with different letters indicate significant differences (*p* < 0.05).

For strawberries exposed to BL during washing, the average values for pH, turbidity and temperature in the strawberry wash water were 3.8, 40 FNU, and 21.5 °C, respectively. These three parameters were not significantly different between BL and no BL groups (Figure 3A,F,G). This suggests that the wash water in both groups was acidic, had suspended solids coming from the shaking of strawberries at 150 rpm, while the temperature of the water stayed stable.

In contrast, BL significantly increased the oxidation reduction potential (ORP) (from an average of 167 mV to 265 mV), and the conductivity (from 100 to 211 µS/cm) of the strawberry wash water (Figure 3B,D), while decreasing the resistivity (from 6 to 0.006 Ω.cm) and percent dissolved oxygen (from 90 to 21%) (Figure 3C,E). This suggests that the wash water had higher concentration of dissolved ionic solids (conductivity), low resistance to electric current (resistivity), low dissolved oxygen percent (DO) which was likely used up to generate reactive oxygen species, and higher capacity to oxidize contaminants (ORP).

Because BL treatment significantly affected certain water physiochemical parameters, correlation analyses were performed between all these parameters and the combined HAV and TV infectivity log reductions calculated for water and strawberries samples. In addition, bacteria count in water or on strawberries were included. Without BL exposure, virus infectivity log reductions on strawberries significantly correlated positively with conductivity and turbidity, while virus infectivity log reduction in water significantly correlated positively with bacteria count in water and dissolved oxygen (Table 1). Under BL exposure, virus infectivity log reductions on strawberries significantly correlated positively with turbidity and virus infectivity log reductions in water (Table 1). The latter significantly correlated positively with conductivity and turbidity and negatively with oxidation reduction potential, resistivity, and bacteria count in water (Table 1).

**Figure 4:**
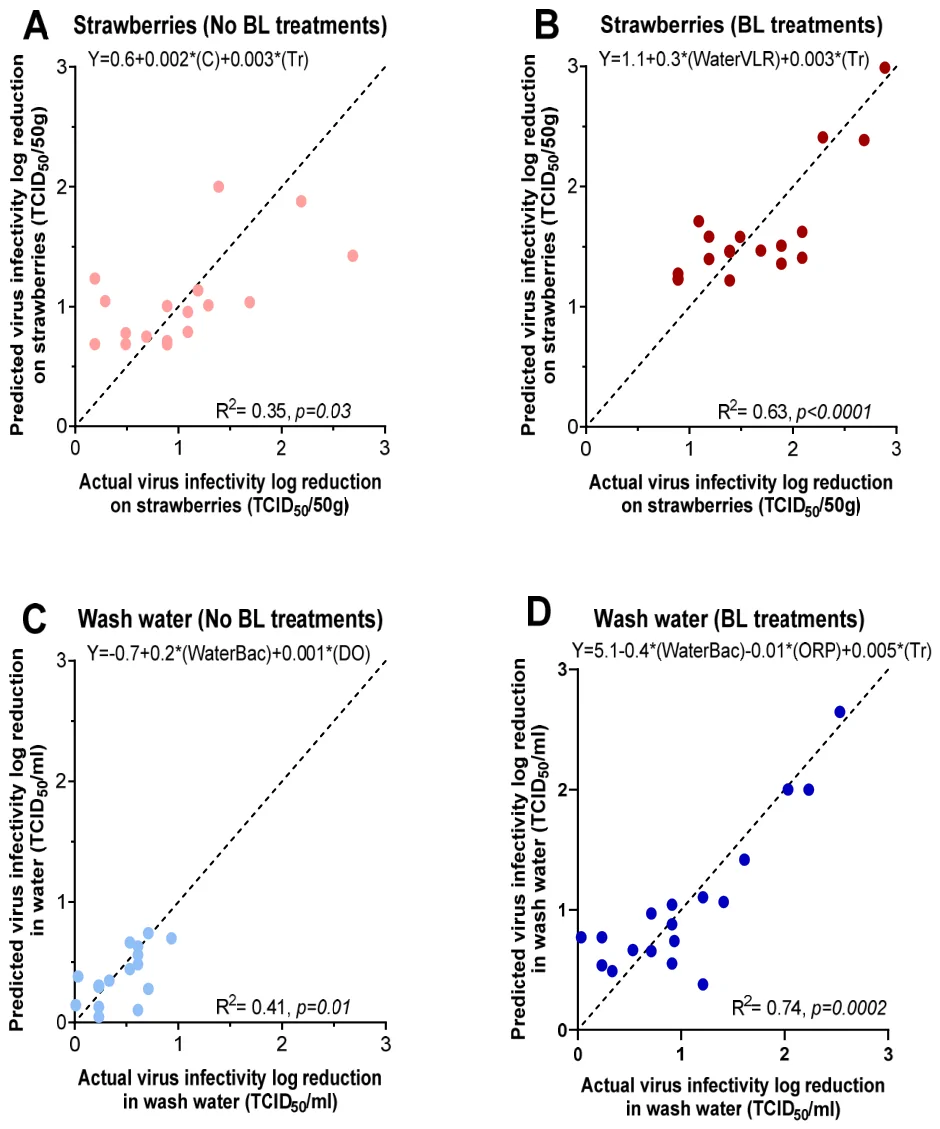
Multiple linear regression for prediction of virus infectivity log reductions on strawberries without (**A**) and with blue light (**B**) as well as in wash water without (**C**) or with blue light treatment (**D**). Dashed line represents line of identity for which predicted values are equal to actual values. Multiple linear regression equations (* indicates multiplication) are shown along with R^2^ and *p* values.

Next, multiple linear regression (MLR) models were fit to the variables that significantly correlated with virus infectivity log reductions on strawberries or in wash water with or without BL treatments (Figure 4). Without BL exposure, the MLR models for virus inactivation on strawberries and in wash water, although significant (*p* = 0.01 and 0.03), exhibited a low R^2^ of 0.35 and 0.41, respectively (Figure 4A,C). This suggested that the predicted log reductions deviated widely from the actual log virus log reductions.

Under BL treatment, the MLR model for virus log reduction on strawberries included two variables namely virus reduction in wash water (Water VLR) and turbidity (Tr) and was extremely significant (R^2^ of 0.63, *p* < 0.0001) (Figure 4B). While the model for virus reduction in wash water initially included all five variables that had strong correlations with virus reduction (shown in Table 1). However, resistivity was eliminated from the MLR model, because it showed significant correlation with conductivity (Pearson r = −0.73, *p* = 0.0005) and oxidative reduction potential (Pearson r = 0.5, *p* = 0.02). Similarly, conductivity was removed from the model, because it correlated significantly with oxidative reduction potential (Pearson r = −0.70, *p* = 0.001) and turbidity (Pearson r = 0.49, *p* = 0.03). The three variables included in the final MLR model for virus reduction in wash water were turbidity, bacteria count in water, and oxidative reduction potential which did not correlate among each other and resulted in highly significant model (*p* = 0.0002) with R^2^ of 0.74 (Figure 4D). Taken together, these preliminary models suggest that predicting virus infectivity log reduction on strawberries under BL treatments is potentially feasible through prediction of virus inactivation in wash water which can be done through easily measured variables namely bacteria count in wash water, oxidative reduction potential, conductivity, and turbidity (Figure 4D). A larger dataset is needed to build better predictive models for virus log reductions on strawberries and in wash water in response to BL treatment.

## 4. Discussion

Blue light offers promising bactericidal and viricidal properties as an alternative or supplementary disinfection strategy [39,47]. To our knowledge, no previous studies examined specifically the effect of BL on enteric viruses on soft fruit matrices such as strawberries during washing. This study explored the effects of BL (405 nm, ~1000 J/cm^2^) treatment against TV and HAV while washing strawberries. A dose of 1000 J/cm^2^ was used based on our previous study that observed significant positive linear relationships between BL doses and virus log reductions on stainless steel surfaces and in sterile and raw surface freshwater [51]. For example, at a BL dose of 1000 J/cm^2^, TV and HAV infectivity was predicted to be reduced by about 0.48 log on stainless steels surfaces. Additionally, generating this BL dose required a 4 h exposure for strawberries in water, which did not cause observable changes in the physical appearance or structural integrity of treated strawberries. Although 4 h treatment exceeds typical industrial strawberry washing times, this BL dose was selected to establish an initial baseline and evaluate the maximum achievable virus log reductions.

Washing is a common method of cleaning strawberries from foreign matter and reducing microorganisms, especially when strawberries are intended for freezing. Typically, a sanitizing solution such as chlorine is used. However, free chlorine’s mechanism of action is pH-dependent and reacts with organic matter, affecting its sanitizing efficiency [52]. Specifically, organic matter generated during washing and the low pH of the strawberry leachate (pH 3–4) greatly reduce the effectiveness of chlorine [24,25]. Free chlorine in wash water is most effective when pH is between 6.5–7.5, whereas strawberry leachate typically has a pH of 3–4 as observed in our study (Figure 3A). Other sanitizers have been proposed such as peroxyacetic acid (PAA) [52]. The general mechanism of action of sanitizers is to create an oxidizing environment to degrade pathogens. An oxidizing environment is where oxidization reactions are more likely to occur and electrons are being transferred from one substrate to another, typically from a molecule to the oxidizing agents. The environment becomes electron-poor as a result and reactive oxygen species largely affect the infectivity of the virus and, to a lesser extent, the integrity of nucleic acids [53]. It was reported previously that when strawberries are washed alone with water or with a sanitizer such as PAA, the natural organic matter from strawberries and the leachate will be released into solution [54]. Leachate from the strawberries consists of various nutrient content such as phenolics, anthocyanins, and even fiber [54]. Our results indicated that washing alone for 4 h caused a ~1.2 and 0.7 log reduction in TV and HAV on strawberries, respectively (Figure 1A,B). Previous studies have investigated washing berries to remove pathogens. For example, an enveloped virus (SARS-CoV-2) spiked on fresh blueberries or strawberries subjected to washing for 10 min caused approx. 1.2 and 1 log reduction, respectively [55]. In the latter study, SARS-CoV-2 dislodged to wash water was found to be infectious which would cause cross-contamination if wash water is not further treated. Another study investigated washing strawberries with potable water for 2 min and found a 1 log reduction in infectious HAV [24]. These studies suggest that some viruses can be dislodged by washing in water alone. Due to matrix effects and other forces such as Van-der-Waals, hydrophobic, and electrostatic interactions, viruses can remain adsorbed onto the berries even during washing. In addition, dislodged viruses can remain infectious in wash water and cross contaminate subsequent clean batches. Consequently, a non-chemical and non-thermal technology is needed to tackle both the issue of viruses adsorbed to berries and to inactivate viruses that end up in wash water.

Blue light treatment alone with a dose of ~1000 J/cm^2^ caused a 0.5 and 0.7 log reduction for TV and HAV, respectively on strawberries (Figure 1A,B). Only one previous study examined the efficacy of BL (405 nm) against TV (4 × 10^7^ titer) spiked (0.5 mL) on the surfaces of blueberries [39]. Specifically, the blueberries were placed on a rotating platform that was illuminated by a high-intensity BL (2 min, 7.5 cm from the blueberries); a custom-built nitrogen gas cooling system blew cold air onto the fruits to mitigate heat generated by BL exposure. However, this approach mitigated the atmospheric oxygen, so it was not used for the blueberry treatment with the photosensitizers. The authors performed 60 pulses for 30 s each, allowing the blueberries to cool for 2 min between each light pulse. At 7.56 J/cm^2^, the authors found a 0.5 log reduction of infectious TV, while the addition of 0.1% of riboflavin to the surface of blueberries caused a 1 log reduction of infectious TV [39]. The riboflavin or rose bengal was a part of the viral cocktail containing infectious TV. A limitation of this study is their use of expensive high intensity, ultra-high-power lamps which necessitated the short treatment pulses and the cooling system built around the blueberries. The high intensity lamps caused thermal heating of the fruit surface up to 32 °C under cooling conditions [39]. Thermal heating of the fruit surface can potentially cause a decrease in fruit quality but also introduce temperature as a confounding factor in virus log reduction. In contrast, in our study the lamps used were low intensity and did not significantly change the temperature of the wash water or affect the physical appearance of the strawberries.

Our results showed that the combination of washing and BL caused 1.7 and 1.5 log reduction for TV and HAV on strawberries. This level of virus reduction obtained in our study is similar to previous studies. For example, a previous study reported limited efficacy of chlorine (200 ppm for 2 min) against MNV-contaminated strawberries, resulting only in 1.2 log reduction [25]. Similarly, another study reported limited efficacy of PAA (80 ppm for 2 min) against MNV-contaminated strawberries, resulting in 1.7 log reduction [56]. Using UV-C light (1300 J/m^2^ for 2 min) during strawberry washing, resulted in 1.3 to 1.7 log reduction in MNV [57]. These chemical- and UV-based systems required minutes to achieve limited virus reductions. In contrast, at the explored BL dose (1000 J/cm^2^ which required 4 h), a similar virus reduction was achieved without using chemicals or UV. However, further optimizations of BL dose, irradiance and addition of photosensitizers are required to achieve faster virus inactivation.

In our setup, it is possible that BL interacted with organic matter generated during strawberry washing to inactivate viruses, thus furthering the idea that the leachates from strawberries promote viral inactivation. Interestingly, exposure to BL also influenced the dislodging of viruses off strawberries (Figure 1). Due to the assumed ROS generated from BL group, these ROS could change the physicochemical characteristics of the water causing more viral elution off the strawberries. Notably, comparing washing alone gave 1.2 log or 0.7 log reduction for TV and HAV, respectively, while washing combined with BL gave 1.7 log or 1.5 log reduction for TV and HAV, respectively. This suggested that there were less viruses on the strawberry, indicating that they were either inactivated on the strawberry (and did not dislodge) or they were eluted off the matrix. This is further supported by examination of the water itself where both viruses were found to have ~0.3 log reduction in the water and with BL treatment had a ~1 log reduction. 

The use of BL during the strawberry wash treatment created an oxidizing environment in the water as evidenced by an increase in the ORP of the water, which may have led to HAV and TV inactivation both in water and on strawberries. This idea is further supported by a previous study that reported an inactivation of 2.6 log of MS2 spiked into water exposed to sunlight when photosensitizers such as rose bengal (0.5 mg/L) were added to the water [58]. While the dose used was high (176 kJ/cm^2^), the spectrum covered the entire UV/visible spectrum, and the authors suggested that the visible spectrum would be able to create ROS within the aqueous environment, thus causing MS2 inactivation [58]. Other studies also observed the inactivation of viruses within a suspension with the addition of photosensitizers; for example causing more inactivation of FCV in DMEM media [41]. The wash water physicochemical parameters suggested that the BL treatment of wash water created an oxidizing environment without changing the pH, turbidity, or temperature of the water (Figure 3A,F,G). This suggested that temperature, turbidity, or acidity (at the noted levels) did not have a direct effect on virus inactivation. Oxidation reduction potential, dissolved oxygen, conductivity did increase with BL treatment (Figure 3B–D). A higher oxidation reduction potential, lower dissolved oxygen, and maintaining the same turbidity seemed to suggest that the BL treatment was able to create ROS from dissolved oxygen which would create a stronger oxidizing environment than no BL treatment. The observed decrease in dissolved oxygen with BL treatment suggests its use as a substrate for the generation of ROS. Previous studies showed that submerging strawberries in water for 4 h would likely result in leaching of secondary metabolites such as anthocyanins which are most prevalent in the outer layers [59]. Organic acids such as malic and citric acids are also expected to leach out of strawberries [60]. The latter is supported by our data showing that wash water became acidic (pH ~3.8) by the end of the washing. Many of these secondary metabolites are thought to absorb wavelength in the UV and visible light spectrum, including blue light [59], suggesting that they would counteract the effect of ROS. However, other fruit secondary metabolites are known to act as photosensitizers [61]. Thus, future studies should examine which strawberry leachate can act as photosensitizer, and how to measure and identify ROS involved to better elucidate the mechanism of BL action on viruses in strawberries and in wash water.

Interestingly, heterotrophic bacteria count on strawberries or in wash water was not reduced upon exposure to BL (Figure 2). Previous studies indicated that BL can negatively affect a wide range of human pathogenic bacteria [47]. Specifically, cocktails of lab-grown human pathogenic bacteria such as *Pseudomonas*, *Staphylococcus*, *Escherichia*, *Listeria* and *Salmonella* when suspended in PBS and exposed to BL (405 nm) at dose of ~1512 J/cm^2^ showed 8, 3.5, 3.2, 2.1 and 1 log. This inactivation is much higher than the no effect observed for BL on strawberry indigenous bacteria at a BL (405 nm) dose of ~1000 J/cm^2^. However, sunlight, including blue light, may have various effects on natural heterotopic bacteria depending on many environmental factors [62], but also on the presence of photoreceptors and protective pigments which allow nonphototrophic bacteria to avoid harmful effects of light [63]. Since our study only accounted for a minor component of the strawberry microbiome, the observed effect may not reflect natural strawberry bacterial communities or foodborne pathogenic bacteria on strawberries. Our results suggest that indigenous bacteria found on strawberries under washing might be tolerant to BL exposure. Similarly, a previous study from our team looked at the effect of BL on indigenous bacteria counts in raw surface freshwater. The effect was minimal, only resulting in ~0.1–0.4 log reduction with various BL doses (130–1520 J/cm^2^) [52]. Indigenous bacteria, like the ones on strawberries and surface freshwater, could be more adapted to environmental stressors, namely the sunlight exposure which consists of both UV and visible light. Because the strawberries used in our experiments were initially grown on farms under natural sunlight prior to harvesting and selling in grocery stores, the bacteria on the strawberries may have already adapted to this stressor, thus limiting the effect of BL on them. 

Multiple linear regression models were fitted to the entire data of water physiochemical parameters, heterotrophic bacteria count in water and on strawberries, to assess whether virus log reductions on strawberries can be potentially predicted or not (Table 1). Highly correlated physiochemical parameters were removed from the MLR models. Without any BL treatments i.e., under washing alone, the models were not ideal with an R^2^ value being 0.35 and 0.41 for strawberries and wash water, respectively (Figure 4A,C). Under washing combined with BL treatments, the models improved to R^2^ of 0.63 and 0.74 for strawberries and wash water, respectively (Figure 4B,D). Prediction of viral inactivation on strawberries can potentially be done from virus inactivation in wash water. However, these MLR are limited because they are built based on a small laboratory dataset, under ideal experimental conditions and have not been validated yet. Therefore, results from these MLR should not be directly extrapolated to diverse industrial wash-water matrices with variable organic loads, turbidity and background microbiota.

A limitation of our study is the lack of measurement of ROS in water. While the data suggested the production of ROS, more investigation is needed to determine the nature and duration of these ROS. Future studies should include other factors such as nutrient content analysis such as phenolic content, anthocyanins, and even fiber (leachate from strawberries). These could act as substrates for ROS; subsequently, more characterization of this needs to be studied. Another limitation of this study is industrial application. For example, the long exposure time (4 h) is not practical in the industry. In addition, a larger dataset is needed to build and validate more robust MLR models. Future studies are needed on the actual pathogen HuNoV and to maximize virus reduction in response to various BL doses, optimize BL irradiance, investigate the effect of exogenous photo sensitizer, and explore engineering options for further scale up industrial application on larger batches of strawberries. Equally important is monitoring the effect of BL treatment on strawberry quality and spoilage microorganisms.

## 5. Conclusions

Blue light treatment while washing strawberries was observed to inactivate infectious TV and HAV on strawberries by ~1.5–1.7 log TCID_50_ and in wash water by ~1 log, respectively. In addition, BL changed certain selected water physiochemical parameters in a way that suggested the production of ROS that could inactivate viruses. The MLR models suggested that viral inactivation on strawberries under BL treatment can potentially be predicted from bacteria count, turbidity, and oxidative reduction potential parameters in the wash water. This study explored the potential use of low-intensity BL treatment, a non-thermal tool, during strawberry washing for virus control on strawberries and in wash water. Further optimization of this technology is needed to offer the food industry-especially in low-resource settings- with a complementary tool for a multi-barrier approach. 

## Figures and Tables

**Table 1:** Correlation analyses between virus infectivity log reductions on strawberries and in wash water with and without blue light treatment and wash water quality physiochemical parameters as well as bacteria counts. Pearson r values are shown. Bold numbers indicate significant negative or positive correlations.

	Virus Infectivity Log Reduction			
	Strawberries		Water	
	−BL	+BL	−BL	+BL
Virus infectivity log reduction in water	−0.12	**0.74**		
Bacteria count in water	−0.13	−0.43	**0.53**	**−0.61**
Bacteria count on strawberry	−0.09	−0.14	0.01	−0.38
pH	−0.09	0.07	0.023	−0.11
Oxidation reduction potential	0.25	−0.31	−0.46	**−0.60**
Dissolved oxygen	−0.27	−0.27	**0.53**	−0.26
Conductivity	**0.53**	0.43	−0.085	**0.50**
Resistivity	−0.22	−0.45	0.45	**−0.46**
Turbidity	**0.50**	**0.75**	−0.082	**0.77**
Temperature	0.34	0.003	−0.31	0.27

## Data Availability

The original contributions presented in this study are included in the article. Further inquiries can be directed to the corresponding author.
